# Meditation and neurofeedback

**DOI:** 10.3389/fpsyg.2013.00688

**Published:** 2013-10-07

**Authors:** Tracy Brandmeyer, Arnaud Delorme

**Affiliations:** ^1^Centre de Recherche Cerveau et Cognition, Paul Sabatier UniversityToulouse, France; ^2^CerCo, Centre National de la Recherche ScientifiqueToulouse, France; ^3^Swartz Center for Computational Neuroscience, Institute of Neural Computation (INC), University of California San DiegoSan Diego, CA, USA; ^4^Institute of Noetic SciencesPetaluma, CA, USA

**Keywords:** meditation, neurofeedback, EEG, biofeedback, enlightenment

Dating back as far as 1957, the academic investigation of meditation and the Asian contemplative traditions have fascinated not only the likes of philosophers and religious scholars, but researchers in the fields of neuroscience, psychology, and medicine. While most of the contemplative traditions are comprised of spiritual practices that aim to bring the practitioner closer to self-actualization and enlightenment, from a neuroscientific and clinical perspective, meditation is usually considered a set of diverse and specific methods of distinct attentional engagement (Cahn and Polich, 2009).

Over the last decade, we have witnessed an exponential increase in the interest in meditation research. While this is in part due to improvements in neuroimaging methods, it is also due to the variety of medical practices incorporating meditation into therapeutic protocols. With the general aim of understanding how meditation affects the mind, brain, body and general health, particularly interesting findings in recent research suggest that the mental activity involved in meditation practices may induce brain plasticity (Lutz et al., 2004).

With its increasing popularity, many people in Western societies express an interest and motivation to meditate. However, for many it can often be quite difficult to maintain a disciplined and/or regular practice, for various reasons, ranging from a lack of time to general laziness. It is possible that machine assisted programs such as neurofeedback may help individuals develop their meditation practice more rapidly. Methods such as neurofeedback incorporate real-time feedback of electro-encephalography (EEG) activity to teach self-regulation, and may be potentially used as an aid for meditation.

While Neurofeedback and Biofeedback have been used since the 1960's, previous neuroscientific and clinical research investigating its efficacy has been limited, lacking controlled studies and significant findings (Moriyama et al., 2012). However, a recent overview of the existing body of literature on neurofeedback research has now led the American Academy of Pediatrics to recognize Neurofeedback, as well as working memory training, as one of the most clinically efficacious treatments for children and adolescents with attention and hyperactivity disorders (ADHD) (Dename, 2013). Neurofeedback has been used to treat a wide variety of other disorders such as insomnia, anxiety, depression, epilepsy, brain damage from stroke, addiction, autism, Tourette's syndrome, and more (Tan et al., 2009; Coben et al., 2010; Cortoos et al., 2010; Messerotti Benvenuti et al., 2011; Mihara et al., 2013). As with all therapeutic interventions it is important to note that individuals who are seeking neurofeedback for diagnostics or for clinical and medical purposes seek qualified and licensed practitioners, as adverse effects of inappropriate training have been documented (Hammond and Kirk, 2008).

Interestingly, many of the conditions that benefit from Neurofeedback treatment are consistent with the conditions that improve with regular meditation practice. For example, both ADHD patients and individuals diagnosed with depression benefit from meditation training (Hofmann et al., 2010; Grant et al., 2013) as well as neurofeedback training protocols (Arns et al., 2009; Peeters et al., 2013). In addition, both meditation and neurofeedback are methods of training mental states. Thus, it is plausible that the mental training involved in meditation may be fundamentally no different than other types of training and skill acquisition that can induce plastic changes in the brain (Lazar et al., 2005; Pagnoni and Cekic, 2007).

One hypothesis to explain the similarity between meditation and neurofeedback is that both techniques facilitate and improve concentration and emotion regulation, for which both attentional control and cognitive control are necessary. When one aims to alter attentional control, one must learn to manipulate the amount of attention that is naturally allocated to processing emotional stimuli. Similarly, when an individual is attempting to exercise or gain some form of cognitive control they must alter their expectations and judgments regarding emotional stimuli (Braboszcz et al., 2010; Josipovic, 2010). These core principles are central to both meditation and neurofeedback, with the distinguishing feature being that meditation is self-regulated, and neurofeedback is machine aided. It is worth noting that the alpha and theta frequency bands trained in most cognitive enhancement neurofeedback protocols (Zoefel et al., 2011) share many similarities with the EEG frequency bands that show the most significant change during the early stages of meditation practice (Braboszcz and Delorme, 2011; Cahn et al., 2013).

The integration between meditation and neurofeedback has already happened in popular culture. Numerous neurofeedback companies already provide so-called “enlightenment” programs to the public. The programs developed by these companies, however, are not all based on the scientific study of meditation and/or neurofeedback, and the reliability and accuracy of signal detection in many of the portable devices currently on the market remains questionable. While many of these companies are relying on the intuitions of their founders for various neurofeedback protocols, it is necessary for these programs to adopt a more rigorous scientific approach, such as those developed for clinical patients being treated using neurofeedback (Arns et al., 2009).

Assuming that reliable and reproducible EEG signatures are associated with specific meditation practices, we may expect that training subjects to reproduce these signatures would support and strengthen their meditation practice. Clinical neurofeedback protocols are aiming toward comparing patients' EEG with large EEG data sets from normal subjects in order to produce a neurofeedback algorithm which rewards subjects (patients) whose EEG becomes closer to that of the normal population (Thornton and Carmody, 2009). Similarly, it might be possible to train users to make their EEG brainwaves similar to the brainwaves of an expert practitioner in a given meditation tradition. Note that we do not argue that the task of the user should be only to up-regulate or down-regulate their EEG. Instead, they would perform a meditation practice and the neurofeedback device would act in the periphery, providing users with feedback on how well they are doing. For this to be feasible, there needs to be a clear identification of the EEG neural correlates of specific meditation techniques and traditions. As evidenced in the literature, there are an abundant number of meditation traditions and styles, many which have vastly differing techniques, methods, and practices. As the mental states associated with particular meditations differ, so does the corresponding neurophysiological activity (Cahn and Polich, 2006). Recent research suggests that complex brain activity during meditation may not be adequately described by basic EEG analyses (Travis and Shear, 2010). Thus, more research and the use of more advanced signal processing tools are needed in order to understand the differences in meditative techniques, and to better define a normative population which EEG brainwaves could be used in a neurofeedback protocol.

Another type of neurofeedback program could help detect mind-wandering episodes. In all of the meditation traditions, practitioners often see their attention drifting spontaneously toward self-centered matters. These attentional drifts are termed mind wandering, and have recently been focused on in neuroscientific research (Braboszcz and Delorme, 2011). Interestingly, in this study on mind wandering, EEG changes in the alpha and theta frequency bands have been observed. A neurofeedback device could provide an alarm to users when their mind starts to wander, therefore supporting and improving upon their meditation practice. Although future research should assess the reliability of these measures to detect single mind wandering episodes, such a neurofeedback system might help support users in their meditation.

Most neurofeedback systems provide auditory or visual feedback that fully engage and demand the attention of the subject. For neurofeedback-assisted meditation, the goal would be to provide subtle cues that do not disturb the subjects' meditation. For example, white noise could be made louder as the subject's EEG departs from the EEG of the normative population of meditators. Similarly, the same white noise amplitude could also reflect the likelihood of the subject's mind wandering. As mentioned earlier, the neurofeedback device would not be a substitute to meditation practice, but rather a means to facilitate and support it in its early to middle states of practice.

Over the last century, and ever more so at present, machines have become extensively integrated into a vast range of human activity. The practice of meditation requires sustained attention that is often hard to achieve for novices, as compared to more advanced practitioners (Brefczynski-Lewis et al., 2003). Therefore, an inspiring application of machine-aided learning may be to help offer alternatives for beginners who struggle with maintaining a regular meditation practice. Learning how to meditate faster and more easily may facilitate access to meditation techniques to a wider audience. Still, it may also be beneficial for more experienced meditators who are interested in deepening their meditation practices. Even the Dalai Lama has publicly stated that he would be the first to use this type of technology, and believes that neuroscience will improve Buddhist practices (Mind and Life Institute, 2004).

This type of application also has the potential of reaching the masses as neurofeedback could be introduced to the domain of smartphones and apps (Szu et al., 2013). In fact, some EEG systems are already compatible with portable and smartphone technology, and it will not be long before we start seeing neurofeedback-based programs for smartphones. Community building over social media using cloud based computing could help users support one another and their meditation practices. In addition to supporting meditation practice, neurofeedback applications can help track the progress of users over weeks and years and assess changes that users may not be consciously aware of, thus encouraging users to pursue their practice. Using neurofeedback to learn meditation truly reflects new, cutting edge science, and via real time feedback we may be able to develop a precise ways to rapidly learn and achieve deeper states of meditation.

In conclusion, it is our belief that mobile neurofeedback systems and protocols that are derived and extend upon meditative traditions and practices offer a promising new direction and platform in mobile technology. These technologies would be not only for people who have taken interest in these kinds of practices or people who have already established themselves in a meditative practice, but for people who are looking for new methods to train, improve, and develop attention and emotion regulation. We want to emphasize that neurofeedback should be used as an aid to meditation while people perform their meditation and not as a replacement to meditation, and that while these devices may aid and assist those in their meditative practices, the goal of these practices themselves is ultimately the decrease of reliance on objects and constructs that provide support. This type of research should also integrate neurophenomenological approaches that take into account first-person reports of subjective experience in conjunction with the experimental investigation of brain activity (Braboszcz et al., 2010; Josipovic, 2010). Real time feedback of brain activity as implemented in neurofeedback may help develop new frameworks for the scientific investigation of embodied consciousness and the interactions between mind and body.
